# Recent neuroscience advances of interest to neurosurgeons, neurologists and neuroscientists — May 2010

**DOI:** 10.4103/2152-7806.63902

**Published:** 2010-05-31

**Authors:** James I. Ausman

**Affiliations:** 69844 Highway 111, Suite C, Rancho Mirage, USA

## USING EACH PERSON'S PERSONAL GENOME TO PREDICT AND PREVENT DISEASE

Can you imagine that in the 21^st^ century, physicians will be examining the genome (complete detailed genetic makeup of DNA sequences) of each patient and then use this data to predict what diseases the patient may have during his/ her lifetime?

In the May 1, 2010, issue of *Lancet* (“Clinical assessment incorporating a personal genome”; Ashley, EA *et al*.; 2010;375:1525-35,), investigators from Stanford University Medical Center reported a detailed genome analysis of a patient with a family history of vascular disease and early sudden death. The investigators took a sample of the patient's blood and used modern rapid techniques for establishing the genetic detail in this patient's chromosomes. They then compared the patient's genetic codes with those of others from large databases to determine what sequences were abnormal in this patient.

They found a number of variations that indicated that the patient, a 40-year-old man, had “a genetic risk for myocardial infarction, type 2 diabetes and some cancers.” They also discovered variants in three genes predisposing the patient to sudden cardiac death. They also found that the patient had a genetic abnormality, which would make him resistant to the antiplatelet action of clopidogrel (Plavix), a drug that might be used to treat thrombosis should he encounter arterial vascular clotting. Furthermore, he had genetic mutants that indicated a hypersensitivity to heparin and that he would need a lower dose if treated with that drug. He was also found to have a tendency to hemochromatosis, which was not clinically evident.

Using a complex scheme developed from studies of others on these specific mutations, the investigators established probabilities for these diseases to occur.

Interestingly, in 2005, another group of investigators used genome sequences to predict which patients would develop complications after surgery (Perioperative Genomics) (Podgoreanu, MV *et al*., J. Am College Cardiol; 2005;46:1965-77). The significance of this work was that the complications we all accept as unavoidable in surgery ARE avoidable once we can sequence the genome in any patient and determine their disease susceptibility.

(*Editor's comment: The Lancet paper is a landmark publication and predicts the future of Medicine in the understanding of disease occurrence and its prevention. Expect this approach to become widespread worldwide, first in the developed world and then in the developing world, in the coming decades. This is an example of the Knowledge Revolution. [See the editorial on “Welcome to the 21^ st^ Century” in this journal.] This medical breakthrough will lead to greater lifespans, and an increasing world population placing demands on food and water resources.*

Fortunately, genetically engineered plants will provide more food. Molecular methods of treating water to make it drinkable will supply the water needs. With the exploration of space, the increased population of the world will find other planets to inhabit [Toffler and Toffler; “Revolutionary Wealth” Doubleday, NY; 2006])

## HOW NF2 TUMORS ARE FORMED — A MOLECULAR EXPLANATION

Neurofibromatosis 2 is an autosomal dominant disease that produces Schwann cell tumors in cranial and spinal nerves. The NF2 gene is located in the nucleus on chromosome 22 at a specifically determined site on the long arm of the chromosome at the position 12.2. The NF2 gene produces a protein called “merlin,” or neurofibromin 2. “Merlin” acts on another gene location in the patient's DNA to suppress that gene's function in promoting cell multiplication and tumor growth. Therefore, merlin acts as a tumor suppressor. If there is a mutation in the NF2 gene, as in people with neurofibromatosis type 2, then a defective “merlin” (neurofibromin 2) protein is produced that does not have tumor suppressor activity, and NF2 tumors then occur. In a paper by Li *et al*. in *Cell* (2010;140:477-90,), the authors found that the “merlin” protein in its closed form, acts in the nucleus as a tumor suppressor. But if the “merlin” protein is transformed to the open form, it loses its tumor suppressor activity and also, in the open form, permits molecular signals to allow cell adhesion and tumor formation. The authors suggest that in the future, the goal should be to find a protein that replaces the defective “merlin” in NF2 patients, which would then act to suppress tumor growth and cell adhesion in patients with NF2. *(As a secondary measure, supplying the patient with the normal NF2 gene would cure the disease permanently. — Editor)*

(This is an outstanding paper that provides a clue to how we will treat tumors in the future. By locating the molecular pathways that are abnormal, molecules can be designed that will target the genetic sites to become tumor suppressors and reverse or stop the disease. — Editor)

## RESTORATION OF INJURED DISCS BY GENE THERAPY: THE FUTURE CURE FOR DISC DEGENERATION?

H. Liang *et al*. received the 2009 Outstanding Paper–Runner Up Award in *The Spine Journal*; 2010;10:32-41, for their paper entitled “Therapeutic effects of adenovirus-mediated growth and differentiation factor-5 in a mice disc degeneration model". According to the authors, disc degeneration affects 60% to 80% of people during their lives in western societies. Disc degeneration pathologically appears as “collapse of the annulus, disc degeneration and loss of proteoglycan content.” Growth and differentiation factor-5 (GDF5) is a protein that is important in the development of the skeletal system. Its deficiency leads to disc degeneration. However, GDF5 addition to chondrocytes (disc cells) can stimulate those cells to produce proteoglycans and collagen, necessary parts of the disc matrix. To get the chondrocytes to manufacture these components required the injection of the gene that produces GDF5 into the chondrocytes. The authors used a viral agent carrying the gene, which infected the chondrocytes and allowed the cells to make GDF5 long term, leading to disc regeneration. Their studies showed that in the GDF5-treated mice, chondrocytes grew from the annulus toward the center of the disc, DNA content of disc (reflecting cell growth) increased, proteoglycan content of the disc matrix increased, and disc height was significantly greater than in the controls, which did not have the GDF5 treatment. The GDF5-treated mice discs were similar to those of normal mice.

(This is an outstanding study. It is an example of how gene therapy will alter the treatments of diseases in the 21^ st^ century. It is also a sign that surgical treatments of disease will not persist, as the advances in molecular medicine will change the therapies that have been developed over centuries from limited scientific knowledge. It is important for neurosurgeons and neurologists to become involved in these types of studies on all the diseases that they treat involving the nervous system and its related structures. This work was done in a department of orthopedics. — Editor)

## WHY DECOMPRESSIVE CRANIOTOMY MAY NOT BE NECESSARY TO TREAT MALIGNANT CEREBRAL INFARCTIONS IN THE FUTURE

Simard, JM *et al*. published a paper on the molecular treatment of malignant cerebral infarctions with massive brain swelling in *Stroke*; 2010;41:531-7, titled “Glibenclamide is Superior to Decompressive Craniotomy in a Rat Model of Malignant Stroke.” The authors note that malignant cerebral infraction occurs in 10% to 15% of stroke patients and is associated with malignant cerebral edema. It usually occurs in patients with large infarctions and carries a mortality of 60% to 80%. It has been treated by decompressive craniotomy (DC); however, this treatment has a higher risk in those over 60 years of age. There is evidence that gene-stimulated (upregulated) sulfonylurea-sensitive membrane receptors (SUR1) are important in leading to cytotoxic edema and the swelling of neurons and astrocytes. The receptor opens a membrane channel, allowing water to enter the cells. A sulfonylurea inhibitor, Glibenclamide, has been found to block these receptors and to limit cerebral edema formation. The authors studied a rat model of middle cerebral artery (MCA) infarction and found that Glibenclamide given 6 hours after the infarction eliminated mortality in the animals and also had a good neurological outcome. In another group of animals treated with decompressive craniotomy, as an alternative to controlling the edema, mortality was reduced to 0, but DC resulted in a poorer neurological outcome for the animals. There was also a control or untreated group of animals for comparison. Histology showed that SUR1 receptors were upregulated (increased) in the capillaries, neurons and white matter after MCA infarction. Also, evidence was obtained indicating that the gene promoter region, which leads to the SUR1 receptor expression and upregulation, was also activated during infarction. Thus infarction leads to a series of genetically controlled molecular events that increase membrane receptors. These receptors during infarction open membrane channels and allow water to enter the cells. The result is malignant edema formation. In animals given Glibenclamide, which blocks SUR1, significantly lesser cerebral edema was seen in the pathology studies when compared with controls. The neurological outcome of the Glibenclamide-treated animals was also better than DC-treated animals. Glibenclamide was given in a low dose that had no effect on blood glucose levels.

(This is also an excellent study, which indicates that by blocking cell membrane receptors on blood vessels and neurons, cerebral swelling can be markedly reduced. Again, knowledge of genetic factors and molecular signaling pathways has led to this discovery that can potentially affect the lives of patients with major cerebral infractions. — Editor)

## AN INTERVENTIONAL MOLECULAR TREATMENT FOR ELIMINATION OF CEREBRAL ANEURYSMS

In the *Journal of Neurosurgery* (2010;112:658-65), Pan *et al*. presented a paper entitled “Embolization of a common carotid aneurysm with rhVEGF coupled to a pH-responsive chitosan in a rat model”. The authors tested a model of delivering liquid molecular agents that obliterate aneurysms in preference to using coils. The key problem is that the liquid agent needs to remain in the aneurysm and then dissolve, as the rhVEGF (recombinant human vascular endothelial growth factor) has a chance to stimulate the production of endothelial cells, fibroblasts, smooth muscle cells and other cells to occlude the aneurysm. Using an isolated rat carotid artery, the authors injected the carrier+rhVEGF mixture into the artery and then removed the proximal ligature so that the mixture would be exposed to blood for 2 weeks before sacrifice. After 2 weeks the pathology and histology studies showed almost complete vessel occlusion in the rhVEGF+carrier model when compared with controls. Also, intimal and luminal proliferation was significantly greater in the treated than in the control animals. Resorption of the chitosan carrier and its replacement by macrophages, smooth muscle cells and collagen were also seen.

(What is needed is a simple method for treating aneurysms that can be used worldwide. This experiment is an example of the kind of work that we will see that will solve this problem. — Editor)

